# Neutrophils, NETs and multiple sclerosis: a mini review

**DOI:** 10.3389/fimmu.2025.1487814

**Published:** 2025-01-28

**Authors:** Moyuan Quan, Huining Zhang, Xiaohong Deng, Huijia Liu, Yanqiu Xu, Xiujuan Song

**Affiliations:** ^1^ Department of Neurology, The Second Hospital of Hebei Medical University, Shijiazhuang, Hebei, China; ^2^ The Key Laboratory of Neurology (Hebei Medical University), Ministry of Education, Shijiazhuang, Hebei, China; ^3^ Key Laboratory of Neurology of Hebei Province, Shijiazhuang, Hebei, China; ^4^ Department of Rehabilitation Medicine, Beijing Zhongguancun Hospital, Beijing, China; ^5^ Department of Internal Medicine, The Military Special Care Hospital of Shijiazhuang, Shijiazhuang, Hebei, China; ^6^ Department of Neurology, The Third Hospital of Shijiazhuang, Shijiazhuang, Hebei, China

**Keywords:** multiple sclerosis, experimental autoimmune encephalomyelitis, neutrophils, neutrophil extracellular traps, therapeutic target

## Abstract

Multiple sclerosis (MS), a chronic inflammatory and degenerative autoimmune disease characterized by the activation of various inflammatory cells, leads to demyelination and neuronal injury. Neutrophils, often underestimated in MS, are gaining increased attention for their significant functions in MS patients and the experimental autoimmune encephalomyelitis (EAE) animal model. Neutrophils play multiple roles in mediating the pathogenesis of autoimmune diseases, and numerous studies suggest that neutrophils might have a crucial role through neutrophil extracellular trap (NET) formation. Studies on NETs in MS are still in their infancy. In this review, we discuss the clinical perspective on the linkage between neutrophils and MS or EAE, as well as the role of NETs in the pathogenesis of MS/EAE. Further, we analyze the potential mechanisms by which NETs contribute to MS, the protective effects of NETs in MS, and their value as targets for disease intervention. NET formation and/or clearance as a therapeutic approach for MS still requires research in greater depth.

## 1 Introduction

Multiple sclerosis (MS) is a chronic inflammatory and degenerative autoimmune disease characterized by demyelination and neuronal injury in the central nervous system (CNS) (1). MS predominantly affects women, and approximately 80% of patients demonstrate a relapsing–remitting course (RRMS) (2). Experimental autoimmune encephalomyelitis (EAE), induced by myelin oligodendrocyte glycoprotein peptide MOG_35–55_, is the commonly used model in exploring pathology and inflammatory response of MS (3).

While most research has traditionally emphasized the involvement of T cells in autoimmune diseases, studies on MS and EAE have shed light on the contribution of various immune cell types to the pathogenesis (1). Among these immune cells, neutrophils, the most abundant leukocytes in humans, have gained recognition for their significant role in both EAE and MS (4). Recent studies have unveiled the previously underappreciated role of neutrophils in autoimmunity (2, 5). They employ various strategies, including phagocytosis, degranulation, cytokine release, antigen presentation and crosstalk with other immune cells, oxidative stress. Additionally, these cells also utilize a lytic form of programmed cell death, leading to the formation of neutrophil extracellular traps (NETs) (5). Exposure to diverse stimuli ultimately results in the breakdown of the neutrophil cytoskeleton, chromatin decondensation, and histone citrullination. Furthermore, it leads to the arrangement of granule contents, such as myeloperoxidase (MPO) and neutrophil elastase (NE), on the DNA scaffold, involving both genomic and mitochondrial DNA (6). However, this process occasionally occurs without compromising the integrity of the cell membrane or causing immediate neutrophil death (6). In addition to eliminating invading microbes (7), NETs are essential for forming central targets in autoimmune diseases (8).

PAD4, a calcium-dependent enzyme responsible for converting arginine to citrulline, exhibits high expression in neutrophils and becomes activated during NET formation (9). The citrullination of histones by PAD4 has the potential to alter their protein structure, disrupting electrostatic interactions with DNA and facilitating chromatin decondensation (9). Moreover, NET is dependent on gasdermin D (GSDMD), which is a pore-forming protein and an executor of pyroptosis. In addition to the crucial function of GSDMD in pyroptosis, GSDMD also plays a vital role in NETs (10, 11). During generation of NETs, GSDMD is activated by proteolysis through neutrophil proteases and subsequently influences further protease activation and nuclear expansion in a positive feedback loop (10). Several studies have shown that suppressing the production of NETs by inhibiting the functions of PAD4 and/or GSDMD provides therapeutic or protective effects in autoimmune diseases (12, 13), suggesting that PAD4 and GSDMD can serve as potential therapeutic targets (11, 14).

In this review, we use the search term of ((Multiple Sclerosis) OR (EAE) OR (Demyelinating)) AND (NETs) and ((Multiple Sclerosis) OR (EAE) OR (Demyelinating)) AND (Neutrophils)) to identify relevant original research articles, case reports, and comprehensive reviews published in English. We systematically exclude studies that do not align with the thematic focus, as well as non-English publications, unpublished research or conference abstracts, thereby ensuring the focus and quality of the review. We discuss the involvement of neutrophils in the pathogenesis of MS and its animal models EAE. Additionally, we addressed the contribution of NETs in MS/EAE pathogenesis, analyzed the potential mechanisms by which NETs contribute to, the protective effects of NETs in MS, and the value as targets for disease intervention. NET formation and/or clearance as a therapeutic approach for MS still requires deeper research. It is necessary to study the heterogeneity of neutrophil and NET and their diverse functions, especially to precisely target their immuno-inflammatory effects while preserving the beneficial properties, such as anti-infection and immune regulation, thereby achieving therapeutic effects in MS.

## 2 A clinical perspective on the linkage between neutrophil and MS

### 2.1 Neutrophils in periphery blood and bone marrow

In recent studies, patients with MS are likely to show elevated counts of neutrophils in the peripheral blood at the early disease stages, before the initiation of acute or chronic treatment at the time of diagnosis (15). Recent research on cellular and molecular features of bone marrow hematopoietic cells in treatment-naïve MS indicated that an increase in myeloid lineage cells including granulocyte-monocyte progenitors, relative to healthy controls. A dynamic production and supply from the bone marrow could be the reason for the significant increase in peripheral blood neutrophils during the MS (16).

In addition, the peripheral blood neutrophils-to-lymphocyte ratio (NLR) has emerged as a promising marker for assessing the systemic inflammatory state and disease process in various autoimmune diseases (17). Several studies have identified NLR as a biomarker in MS and have examined its relevance as a potential marker for disease progression (18–21). A previous study has shown that an elevated NLR is associated with disease process at onset in RRMS patients (18). Furthermore, NLR effectively discriminates progressive and relapsing status in MS, with a higher NLR independently linked to neurological disability, an increased rate of relapse, and predicting the necessity of treatment escalation (19–21).

Neutrophils are primed in periphery blood of individuals with MS and display an activated phenotype (3, 22). A notable expansion of granulocytes, particularly CD15^+^ neutrophils, is observed in inactive RRMS, accompanied by a reduction in the lymphocyte population (2). This finding holds potential for robustly distinguishing between different types of MS courses and could potentially aid in optimizing disease-modifying therapy (DMT) monitoring. Another study observed a significantly higher expression of TLR-2, a pro-inflammatory receptor, on peripheral blood neutrophils of patients with MS, suggesting their potential role in the pathogenesis of the disease (23).

In the blood of MS patients, increased concentrations of neutrophil-activating chemokines, granulocyte recruiting chemokines, and neutrophil-derived enzymes are detected (e.g., CXCL1, CXCL8, CXCL5, NE, MPO) and these molecules are associated with the formation of new inflammatory lesions and correlated with measures of MS lesion burden and clinical disability (24, 25).

These studies indicate the expansion of neutrophils in peripheral blood along with phenotypic and functional pro-inflammatory alternation, suggesting their involvement in the pathogenesis of MS. Furthermore, research has indicated that peripheral immune responses targeting the CNS occur early in the disease process, while immune reactions within the CNS dominate later (23).

### 2.2 Neutrophils in CNS

Pediatric patients with MS exhibit the presence of neutrophils in their cerebrospinal fluid (CSF) (26). Specifically, adult patients with RRMS also have an increased frequency of IL-11R^+^ neutrophils in their CSF compared to matched healthy controls (27). In the early stage of MS, a noteworthy correlation has been observed between the expansion of neutrophils in the cerebrospinal fluid and the levels of IL-17A (28).

Molecular investigations have identified the presence of mRNA derived from the neutrophil-specific protein ASPRV1 in brain lesions from MS patients (29). Notably, these mRNA levels are prominently elevated in severe cases of MS compared to mild or moderate forms, as well as in normal-appearing white matter (29). In addition, similar to peripheral blood, the levels of many neutrophil-related molecules (CXCL1, IL-1) also increase in the brain (30) and CSF of MS patients (31). These findings provide indirect evidence of the role played by neutrophils in the central nervous system (29).

However, previous studies have shown that neutrophils are not a pronounced pathological feature in MS CNS tissue sections (4), and cerebrospinal fluid neutrophils decrease with disease duration, although they are recruited to the CNS early in the inflammatory process before disease onset (32). This does not necessarily exclude a role for neutrophils in MS. It is challenging to demonstrate that neutrophils exhibit phenotypic plasticity, concurrently expressing typical markers associated with both macrophages and dendritic cells, as elaborated earlier (2). This makes it difficult to differentiate neutrophils from other myeloid cells through conventional histological techniques. Furthermore, it is worth considering that neutrophils may primarily exert their influence on MS pathogenesis through peripheral mechanisms rather than within the CNS (33). An alternative hypothesis suggests that neutrophils may infiltrate the CNS transiently or at an early stage, thereby manifesting their effects. Samples obtained from autopsies of individuals with this chronic, multiphase disease do not capture the potential transient contribution of neutrophils in the initial formation of CNS lesions. These factors contribute to the frequent oversight and underestimation of the crucial role of neutrophils in the pathogenesis of MS. Moreover, focusing on the various clinical stages of MS, the heterogeneity of neutrophils, and their regulatory functions within the peripheral immune system is particularly significant.

## 3 Evidence for the Implication of Neutrophils in EAE

### 3.1 Neutrophils in bone marrow

The hyperresponsiveness of bone marrow in EAE mice resembles that in MS patients (16). Additionally, the numbers and activity of neutrophils were robustly increased in bone marrow of femurs and CNS-surrounding bones in EAE mice with actively or passively transferred disease (16). The results demonstrate that newly generated neutrophils can be mobilized from bone marrow and penetrate the CNS tissues, suggesting that neutrophils may contribute to neuroinflammation during EAE development (16). The study illustrates the possible the source and origin of neutrophils expanded in MS and EAE.

### 3.2 Neutrophils in periphery blood and CNS

Neutrophil counts are elevated in both circulating and CNS immediately during the preclinical stage and at the onset EAE (25, 32, 34). Moreover, in EAE mice, neutrophils had already appeared within the meninges and perivascular inflammatory foci shortly before the onset of clinical symptoms, and their numbers continued to rise as EAE progresses (35, 36). At the peak phage of EAE, neutrophils were observed in brain and spinal cord within areas of vascular leakage, demyelination, and axonal loss (37, 38).

Atypical EAE is associated with brain inflammation and characterized by ataxia and tremors. A previous study has reported that the brain-targeted atypical EAE is dependent on preferential neutrophil infiltration in the cerebellum (39, 40). Aging also impacts the MS brain, rendering it more vulnerable to gray matter injury (41). Age-dependent gray matter demyelination is associated with leptomeningeal neutrophil accumulation (42). Intracranial inoculation of the neuroadapted JHM strain of mouse hepatitis virus (JHMV) into susceptible strains of mice results in demyelination similar to MS. This model in which expression of CXCL1 is under the control of a tetracycline-inducible promoter active within GFAP-positive cells results in sustained neutrophil infiltration in the CNS that correlates with an increase in spinal cord demyelination (43). Additionally, a noteworthy reduction in disease severity and prevention the development of clinical EAE is observed when neutrophils are depleted prior to disease initiation (44, 45). These observations indicate that neutrophils play a role in promoting inflammation during the initiation and advancement of EAE, as well as contributing to demyelination and axonal damage in CNS during the acute phase of the disease.

Inhibition of neutrophils migration to CNS significantly alleviated EAE onset and severity (39). Inhibition of neutrophil migration by depletion of the neutrophil chemokine receptor CXCR2 (39, 45) significantly ameliorated the onset and severity of EAE. CXCR2 affects cuprizone-induced demyelination by enhancing the effector functions of infiltrated neutrophils in the CNS (46). Moreover, signaling to CXCR2 governs a broad array of neutrophil effector functions, including degranulation and gene expression. CXCR2 in neutrophils is critical in triggering CNS neuronal damage via ROS generation, which leads to prolonged EAE disease (47). Additionally, GM-CSF similarly contributes to the mobilization of neutrophils during inflammation. In the preclinical phase of EAE, there is an accumulation of GM-CSF which correlates with an increased neutrophil count observed in CNS and bloodstream, and GM-CSF(-/)- mice are resistant to EAE (48–50). Moreover, claudin-5 which is a major tight junction protein of the microvascular endothelium comprising the blood–brain barrier (BBB), appearance on leukocytes plays a key role in leukocyte trans-endothelial migration into the CNS (51). Of all the leukocyte subtypes, neutrophils achieve the highest percentage of CLN-5^+^ (51). Several leukocyte subtypes, mainly including neutrophils variably acquire claudin-5^+^ in blood before they enter the CNS, representing a novel mechanism to guide across the BBB during progression of EAE (51).

### 3.3 Neutrophils in Lymph node

Immunological episodes in MS are initiated in CNS-draining lymph nodes (LNs), where immune cells first become activated and sensitized to myelin antigens (52, 53). The frequency and numbers of neutrophils in the secondary lymphoid organs was increased (54). Another study arrived at a similar conclusion, observing the early accumulation of neutrophils within lymph nodes shortly after EAE induction (55). Neutrophil migration to draining LN is a key amplifier of autoreactive CD4^+^ T cells expansion locally, and subsequently of the severity of EAE, in a manner that is controlled by TLR9 signaling (55). Selectively repressing the expansion of mature, activated CD11b^+^Ly-6G^+^ neutrophils in the peripheral lymphoid compartment and their influx into the CNS during the induction phase of chronic neuroinflammation in EAE (54).

### 3.4 Neutrophils in Dorsal root ganglion

In addition to its role in immune function regulation and involvement in pathogenic mechanisms, research has revealed that neutrophils also contribute to the occurrence of accompanying symptoms in MS. Pain is a frequent and disabling symptom in patients with MS; however, the underlying mechanisms of MS-related pain are not fully clarified.

A previous study has shown that after MOG35-55 immunization, neutrophils immediately accumulated in the dorsal root ganglion (DRG) of EAE mice (56). TLR4–CXCL1 pathway in DRG neurons triggers neutrophil recruitment in the DRG and subsequent mechanical allodynia in response to MOG35–55 (57). Adoptive transfer of MOG35-55-stimulated wild-type neutrophils into the dorsal root ganglion induced mechanical allodynia in the recipient mice (56).

Moreover, increased cathepsin E in neutrophils contributes to the generation of mechanical allodynia through promoting the production of elastase (56). Inhibition of Cat E-dependent elastase production in neutrophil might be a potential therapeutic target for pain in patients with MS.

### 3.5 Neutrophils in Gut

MS has been reported to be associated with intestinal inflammation and gut dysbiosis (58, 59). Gut recruitment of neutrophils is associated with the development of gut dysbiosis and intestinal inflammation (58). Increased neutrophil infiltration in the intestinal tissue concomitant with IL-17 expression and myeloperoxidase activity was found to correlate well with EAE clinical activity (60). Furthermore, fecal Lipocalin-2 (Lcn-2), a biomarker of intestinal inflammation, was significantly elevated and predominantly produced by gut-infiltrating neutrophils (61). The elevation of fecal Lcn-2 levels correlated with reduced bacterial diversity and increased levels of neutrophil elastase and calprotectin. This suggests that neutrophils through secreting Lcn-2 are involved in the pathogenesis of MS by disrupting gut microbiota equilibrium and exacerbating intestinal inflammation.

## 4 NETs involvement in MS/EAE

Studies have demonstrated that neutrophils play multiple potential roles in MS/EAE, such as phagocytosis, degranulation, cytokine release, antigen presentation and crosstalk with other immune cells, oxidative stress, and more (3, 4). This review primarily focuses on the NETs.

NETs are elevated in the serum of MS patients compared to healthy controls, probably due to the chronic inflammatory environment that primes neutrophils (22). Remarkably, the elevated plasma levels of NET-associated proteins, such as NE, MPO and DNA-MPO complexes in MS patients, correlate with clinical disability and lesion burden (24, 25, 62). In addition, PAD4 which highly expressed in neutrophils, is essential for the formation of NETs by promoting chromatin decondensation through histone citrullination (63). PAD4 were upregulated in RRMS (64), and PAD4 inhibitor exhibited improved clinical outcomes in the EAE mouse model and in the cuprizone-mediated demyelination model (65, 66).

Various studies have indicated that only a specific subtype of neutrophils participates in NET formation, particularly during inflammation in the absence of pathogens, implying diversity within the neutrophil population (67). A distinct subset of neutrophils, known as low-density granulocytes (LDGs), exhibits a heightened tendency for NET formation compared to their normal density counterparts (68). Interestingly, CD14^-^/CD15^high^ LDGs were notably more prevalent in peripheral blood of MS patients than in healthy controls (69). This provides indirect evidence for the involvement of NETs in the pathogenesis of MS.

Furthermore, gender−specific disparities were found in circulating NETs; their degradation and NET formation were observed in patients with relapsing-remitting MS, suggesting that NETs may contribute to gender−specific characteristic in the pathogenesis of MS (70).

During EAE progression, DNase-1 was used to dissolve the accumulated NETs. The DNase-1-treated group achieved a lower disease score and experienced lesser weight loss and mitigated demyelination (71). Neutrophils significantly infiltrate the CNS and form NETs, which are modulated by NLRP3 (71). NLRP3 facilitates NET formation in a ROS-dependent and PAD4-independent manner in brain-infiltrated neutrophils (71). In addition, PAD4 inhibitors effectively improve neurological symptoms in demyelinated mice by inhibiting the formation of NETs (65).

These studies shed light on a solid association between the pathogenesis in MS/EAE and NET formation. However, the specific mechanism is still far from being elucidated.

## 5 The potential mechanisms of NETs in MS/EAE

### 5.1 The regulation effects of NETs on other innate and adaptive immune cells

Recent studies have emphasized the significance of NETs in autoimmune disorders, particularly their capacity to regulate the activation and differentiation of other innate and adaptive immune cells, playing a pivotal role in both the onset and progression of MS and EAE.

IL-17-producing Th17 cells play a key role in MS. Previous research has demonstrated that NETs, through their histone protein components, directly activate T cells, particularly promoting Th17 cell differentiation. This regulatory function of neutrophils, NETs, and their histones is mediated downstream of TLR2 in T cells, leading to STAT3 phosphorylation (62). In addition to modulating Th17 cell differentiation, NETs can also directly regulate T cell activation by enhancing the expression of CD69 and CD25 (72). Moreover, NLRP3-supported NET formation exacerbates disease severity through triggering Th1 and Th17 cell recruitment in CNS (71).

NETs directly activate autoreactive B cells by interacting with TLR9 receptors through NET DNA complexes (73). Additionally, NETs can bridge the innate and adaptive immune systems by inducing the secretion of the B-cell-activating cytokine BAFF (74).

Within the adaptive immune system, T cells play a vital role in recognizing specific antigens presented by antigen-presenting cells (APCs) through major histocompatibility complex (MHC) molecules. NET components possess the capability to activate APCs, including dendritic cells (DCs), through Toll-like receptors (TLRs), thereby triggering the maturation of DCs and leading to Th1 cells polarization (75). Coculturing bone marrow-derived macrophages (M0 and M2) with high concentrations of NETs led to the significant secretion of proinflammatory cytokines and the upregulation of M1 markers, while conversely, downregulating M2 markers (76). Moreover, decondensed DNA present in NETs resulted in the activation of the macrophagic cytosolic cGAS-STING pathway which ultimately results in the amplification of the initial immune response (77).

**Figure 1:**
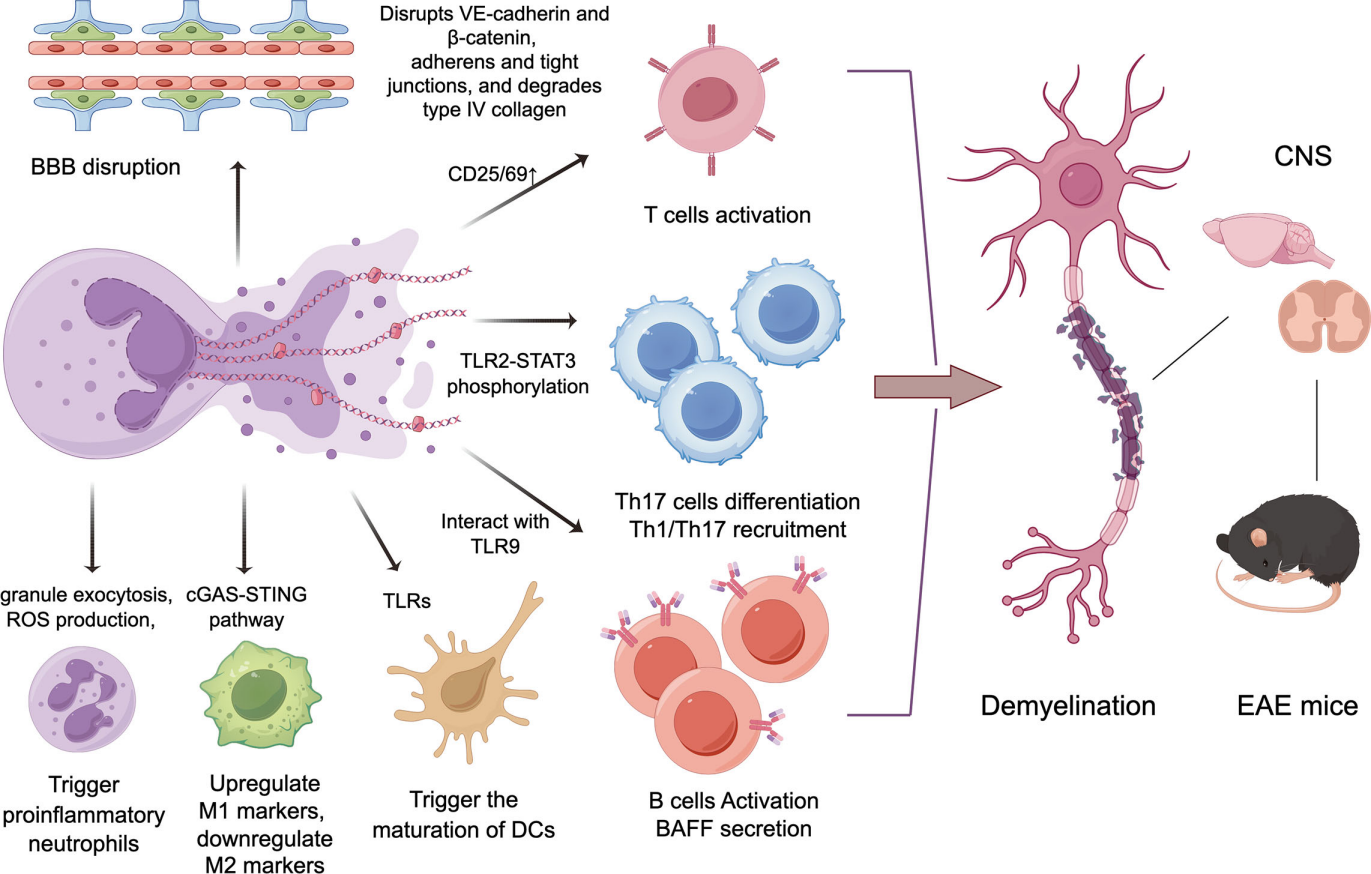
The potential mechanisms of NETs in the progression of MS/EAE. By disrupting the blood-brain barrier, inducing proinflammatory neutrophils, and activating other adaptive and innate immune cells including T/B lymphocytes and myeloid immune cells, NETs can trigger demyelination in the central nervous system, then leading to neurological deficits in MS and EAE.

These experimental results indicate that NETs can regulate immune responses by interacting with both innate and adaptive immune cells (**Figure 1**), thereby participating in the pathogenesis of MS and EAE.

### 5.2 NETs activate proinflammatory functions of Neutrophils

NETs were isolated from cultures of PMA-exposed neutrophils. Exposure to these isolated NETs activated several neutrophil functions, including granule exocytosis, reactive oxygen species (ROS) production, and NOX2-dependent NET formation (74). NETs trigger proinflammatory functions in neutrophils, suggesting a self-amplifying mechanism in inflammation. This NET-induced amplification may also contribute to autoimmune conditions like MS.

### 5.3 BBB disruption

Under healthy conditions, neutrophils have difficulty crossing the BBB, so they are rarely found in the normal brain. It has been suggested that cytotoxic components of NETs may contribute to BBB damage. Activated neutrophils release neutrophil elastase (NE)—possibly within NETs—which disrupts adherens junction proteins VE-cadherin and β-catenin, increasing BBB permeability (78). NET-associated MMP-9 in the cerebral microvessels degrades type IV collagen in the basal lamina, compromising BBB integrity (79). Additionally, histones also increase BBB permeability by disrupting adherens and tight junctions (80).

## 6 Potential protective Function of NETs in MS/EAE

Amyloid fibrils are effective therapeutics for EAE. They induce neutrophils to release NETs, reducing their numbers in the blood, and stimulate plasmacytoid DCs to secrete type 1 IFN (81). A study suggests that NETs promote Treg differentiation and induce periphery tolerance (75).

Another study indicated that FTY720, an immunosuppressive agent widely used for treating MS, potently promoted the rapid formation of NETs (82) independent of NADPH oxidase (NOX) (83). Pharmacological inhibitor experiments indicated that that FTY720-induced NETs depended on the activation of p38 and AKT (83). In contrast, the citrullination of histone H3 and PAD4 did not mediated FTY720-induced NET formation (83). Collectively, these findings indicated that NETs induced by FTY720 could protect MS patients against pathogen infection (83). The impact of NETs on MS and EAE may depend on the balance between their pro-inflammatory function and their potential antibacterial role.

## 7 NETs as a possible target for biomarkers and therapies in MS/EAE

Critical enzymes essential for NET formation have been identified and targeted for therapeutic interventions (84). Among these enzymes, PADs play a crucial role in mediating NET formation by facilitating chromatin decondensation. Increased enzyme activity and overexpression of PADs have been observed in MS (64, 85, 86). Recent advancements include the discovery of new selective PAD4 inhibitors that bind to a calcium-deficient form of the PAD4 enzyme. These inhibitors have validated the critical enzymatic role of both human and mouse PAD4 in histone citrullination and NET formation (87). Investigation into hypercitrullination, specifically involving PAD2 and PAD4 enzymes, has identified them as relevant potential targets in the context of MS (86, 88, 89). Furthermore, a recent study has demonstrated that compounds designed as potential inhibitors targeting PAD enzymes exhibit significant efficacy in both the MOG35-55 induced EAE mouse model and the cuprizone-mediated demyelination model (65, 90, 91). This collective evidence underscores the therapeutic potential of targeting PAD enzymes to modulate NET formation in the context of MS.

The gasdermins (GSDMDs), family of pore-forming proteins, are emerging key regulators of innate immunity and autoinflammatory disorders (92). Multiple studies have recently characterized their crucial roles in NETs generation (92). In a recent study, GSDMD was identified as a driver of EAE in mice (93). This finding represents the first indication of a possibly essential role of GSDMD in the pathogenesis of MS. Consequently, targeting NETs through GSDMD might be a potential therapeutic strategy for MS treatment.

Currently, various disease-modifying therapies are widely used in the treatment of MS during remission. Although none of them work directly by affecting NET formation, several drugs do influence the formation of NETs, which might be an additional mechanism through which these drugs exert their effects. A study has revealed that dimethyl fumarate, a commonly used DMT in MS, could inhibit NET formation and alleviate neutrophil-mediated chronic inflammatory diseases (94). Bruton’s tyrosine kinase (BTK) became an appealing therapeutic target for MS. In addition to its established role in B cell biology, BTK was also found to be indispensable for neutrophils. An other recent study revealed that BTK inhibitors (evobrutinib, fenebrutinib, and tolebrutinib) reduce release of neutrophil extracellular traps and neutrophil activation (95). Increasing evidence suggests that inhibiting NETs formation can alleviate MS or provide protective effects in EAE, making NETs a promising therapeutic target.

## 8 Concluding remarks and future directions

Increasing evidence indicates that neutrophils play a significant role in MS and EAE. An increase in neutrophils has been observed both centrally and peripherally in MS patients. Under disease conditions, peripheral neutrophils exhibit an activated phenotype, which helps distinguish different MS courses. Additionally, NLR as a promising biomarker, they are closely associated with MS activity and neurological disability. Although neutrophils are not a prominent pathological feature within the CNS in MS, the increase in neutrophils and their related proteins indicates their involvement in the pathogenesis of MS. In EAE mice, increased numbers and enhanced activity of neutrophils have been observed in multiple tissues including bone marrow, peripheral blood, CNS, lymph nodes, dorsal root ganglia, and gut. These neutrophils contribute to the amplification of immune-inflammatory responses, pain, and the occurrence of intestinal inflammation.

Although these studies confirm the involvement of neutrophils in the pathogenesis of MS/EAE, there is another hypothesis based on research on EAE suggesting that neutrophils have a protective role. One study showed depleting Ly6G+ cells that there was an increased accumulation of B cells in the CNS, which promoted the progression of EAE, indicating neutrophils beneficial effect (96).

Therefore, investigating the functional heterogeneity of neutrophils is crucial, as their varying cellular functions may play markedly different, and even opposing, roles in the onset and progression of central nervous system demyelinating disease. A recent study identified three types of activated/netting neutrophils in NMOSD lesions, with CitH3+DAPI+MPO+ neutrophils/NETs featuring extracellular non-filamentous structures and CitH3+DAPI+MPO+ neutrophils lacking extracellular structures being predominant in early active lesions ^(^ 97). However, neutrophils were absent from MS lesions, we can still observe neutrophil heterogeneity in the CNS-draining lymph nodes. Our previous research has indentified three distinct subclusters of neutrophils in cervival lymph node of EAE mice (98). The proportions of each subcluster differ between the acute and chronic phases of EAE, suggesting that each plays a different role. GO analysis of the neutrophil subclassifications revealed that subcluster 1 is enriched in processes such as antigen processing and presentation (98), Subcluster 2 in leukocyte migration and subcluster 3 showed enrichment in regulation of reactive oxygen species metabolic process and NETs generation. Given that neutrophils exhibit functional heterogeneity and depleting neutrophils is not practical in most human conditions, researchers are now concentrating on identifying specific functions of neutrophil subsets that can be effectively targeted in treatments.

The discovery that NETs form in autoimmune conditions and can worsen the disease has made them highly intriguing targets for clinical intervention. NETs and NET-associated proteins are elevated in the serum of MS patient. A study has shown that NETs may contribute to gender-specific characteristic in the pathogenesis of MS. Moreover, infection with ability to form NETs could be a possible environmental factor associated with the onset or relapses of central nervous system demyelinating disease (99). In EAE, destabilization of already formed NETs, or inhibiting formation of NETs alleviates EAE and neurologic pathology. The research illustrated that NETs are involved in the pathogenesis of MS/EAE. The potential mechanisms involve interactions between NETs and other adaptive and innate immune cells to amplify immune cascade responses, activate neutrophils, and disrupt the BBB. An increasing body of literature supports a role for neutrophils as players in the orchestration of innate and adaptive immunity (100). During acute and chronic phage of EAE, neutrophils rapidly migrate to CNS draining lymph nodes, where they engage bidirectional interactions with B‐ and T‐lymphocyte and myeloid immune cells (98). The specialized immunoregulatory properties and mechanisms of distinct neutrophil populations, originating under pathological conditions and targeting B/T cells and other immune cells, should be more extensively studied.

Recent progress in understanding NETs is expected to lead to new therapeutic strategies that will benefit patients. PAD4 and GSDMD both play important roles in the formation of NETs. Inhibiting the functions of PAD4 and GSDMD can suppress the generation of NETs, making them potential therapeutic targets. Beyond PAD4 and GSDMD inhibitors, future therapeutic strategies might involve the development of anti-citrullinated protein antibodies to prevent NET formation. Additionally, dimethyl fumarate and TBK1 inhibitor, can also inhibit the formation of NETs, thereby expanding the mechanisms through which this drug exerts its effects in MS. In addition to inhibiting the formation of NETs, targeting treatments might also include drugs that promote the degradation of NETs to treat MS. However, the antibodies and drugs still require extensive research, and the function need to be validated in EAE mice.

The neutrophil subset closely associated with NETs formation represents a highly promising research direction, as it could allow for precise targeting of this subset while minimizing the impact on other neutrophil functions. Low-density neutrophils are considered more susceptible to form NETs. Moreover, CD177 is a glycoprotein that is exclusively expressed on neutrophils, and CD177^+^ neutrophils release more ROS, MPO, and calprotectin, and produce more NETs than CD177^–^neutrophils inflammatory bowel diseases (IBD) (101). However, accurately identifying the characteristics or markers of the neutrophil subset most closely linked to NETs formation remains a significant challenge. Further investigations on the interaction between NETs/NETs-generated neutrophils and other immune cells may lend additional insights to the development MS, as well as provide potential therapeutic strategies.

Studies investigating NETs in MS/EAE thus far do not account for NET heterogeneity; the specific types of NETs is rarely reported. NETs embedded with mitochondrial DNA (mtNETs) have been described as early-forming, non-lytic, and dependent on ROS. These NETs were found to contain mitochondrial DNA along with granule proteins such as NE and MPO but, interestingly, not genomic DNA. mtNETs have now been implicated in a range of autoimmune and inflammatory diseases including SLE and IBD (101, 102). NETs also play an important role in resisting infections, therefore it is clear to see that the different compounds that inhibit or clear NETs may have other unwanted effects. Therefore, if NETs are to be considered a future therapeutic target in MS and other immune diseases, they need to be studied in greater resolution and it is necessary to preserve their protective, anti-infective functions.

Our current understanding of the biological characteristics of neutrophils and NETs in MS/EAE is still limited, and even the detection of marker is in its infancy. NET components, subsets, and the presence of NET generation may represent potential biomarkers/indicators useful for diagnosing of disease process in MS.
